# Restaurant Inspection Reports as a Proxy Measure for Occupational Health and Safety: South Asian Restaurant Workers in New York City

**DOI:** 10.29024/aogh.2332

**Published:** 2018-08-31

**Authors:** Ismail Nabeel, Hasanat Alamgir

**Affiliations:** 1 Department of Environmental Medicine and Public Health, Icahn School of Medicine at Mount Sinai, 17 East 102nd Street, New York, NY, US; 2 Department of Health Policy and Management, New York Medical College, Valhalla, NY, US

## Abstract

There is a great need to develop workplace health and safety surveillance systems for small businesses to systematically understand the cause, nature, and severity of injuries and illness of their workers. Restaurants can be hazardous workplaces for the nature of the business, materials handled, and tasks completed. Some of the traditional South Asian establishments/restaurants rely heavily on the traditional way of food preparation. Workers in these places may work in less than ideal conditions with minimal or no workplace health and safety regulations or programs. We have explored a unique idea of using NYC’s restaurant inspection reports as a possible surveillance tool using the overall restaurant grade and specific violations. Findings show 19% of the Indian, 26% of Bangladeshi, and 15% of Pakistani restaurants did not achieve grade A in these inspections suggesting that around 20% of these restaurants workers are more likely to work in a relatively hazardous or unhygienic working conditions. Using restaurant inspection grade as a proxy measure for employee safety and working conditions may prove to be a useful and practical measure for such an industry.

During the past few decades, public awareness and interest in the USA has steadily increased over the working conditions at formal and informal sectors in the developing countries. This phenomenon may have resulted from a combination of heated political debates about jobs leaving this country, awareness about exploitation of cheap labor overseas and worker rights repression, and news stories on frequent fatal factory fires and factory building collapses in other parts of the world. While making goods that are coming to the western consumers at a low price, workers in many of these outsourced industries are working in deplorable conditions, working very long hours at barely living wages with limited or no access to standard employment conditions and benefits. However, there are millions of immigrant workers, both documented and undocumented, working in somewhat comparable conditions within the USA and there is inadequate knowledge, evidence, awareness, and research on these workers’ health, safety and wellness issues who are living and working right in our backyard.

In 2013, the number of foreign-born individuals in the US was 14% of its total population [1]. With almost 46 million individuals, this makes the US the nation with the largest number of migrants. A major destination for these immigrants is New York City (NYC) and the largest population group is South Asia: India, Pakistan, and Bangladesh. Nearly 500,000 immigrants from India, Pakistan, and Bangladesh now live in NYC and its vicinity [2]. They prefer to settle here for availability of jobs and for family and social connections. A large proportion of them with low fluency in English, lower educational attainment and job-skills settle in lower socioeconomic neighborhoods in this very large city and often start to work as taxi drivers or in grocery stores, liquor stores, convenience stores, bars, restaurants and gas stations, or in other family owned businesses. These smaller establishments are critical to the economy of NYC and often serve as the primary source of low-wage jobs for many newly arrived unskilled immigrants.

Previous studies on immigrant workers have largely focused on immigrants from Latin America and a few studied Chinese, Vietnamese or other East Asian workers [3,4,5,6]. The findings highlighted the additional health burden for these ethnic minority population from unsafe and unhygienic workplaces. The cost of receiving health care was reported to be crippling for these immigrants – both documented and undocumented. Integration into the mainstream economy often takes a long time for many immigrants. One study on Chinese-Americans noted that health problems are aggravated by language and cultural barriers in seeking medical care. Many Chinese immigrants are poorly informed about the availability of services or find existing facilities to be inaccessible because of their poor English literacy. Others may have poor understanding of Western medicine and still adhere to ancient Chinese remedies [3]. Another study reported that immigrants were primarily employed in jobs that required limited skills and offered few benefits. Only 20% participants had employment-related health insurance, and almost 60% did not have workers’ compensation coverage [4]. The disparity in health care benefits may be less a function of immigrant status than of the types of jobs held by these workers. We found no study focusing on South Asian immigrants working in NYC’s restaurants.

Restaurants can be hazardous workplaces for the nature of business, materials handled, and tasks completed. Working with hot equipment, oil, sharp knives, lifting heavy objects, working in hot work areas for a long period of time and on slippery floors or floors cluttered with objects may result in muscle strains, sprains, and tears; cuts and lacerations and burns and scalds [7]. OSHA reported 185,200 cases of nonfatal occupational injuries and illnesses with an incidence rate of 3 per 100 full-time workers for Restaurants and other eating places (Private Industry) in 2015 [8]. However, OSHA’s inspections and enforcement of the regulations may be limited for many of these ethnic restaurants which are often run by family members, supported by underage children of the owners, employ casual or part-time workers, and workers without formal contracts. Also, many have 10 or fewer employees making them exempt from OSHA injury and illness recordkeeping.

Some of the traditional South Asian establishments/restaurants rely heavily on traditional way of food preparation to create more authentic taste of their cuisine. Different types of ethnic cooking apparatus such as ‘kiln oven’ or ‘tandoor’ are used to prepare the traditional bread (chapati, roti), which may contribute to unique work-related hazards and injuries specific to these ethnic establishment.

Workers in these restaurants likely work in less than ideal conditions with minimal or no workplace health and safety regulations, programs, policies, or practices. Given the scarcity of evidence and difficulty in accessing these populations, we have explored a unique idea on how to shed some light on their vulnerability using NYC’s restaurant inspection reports as a possible surveillance tool (Table 1 and 2).

**Table 1:** Restaurant Grades in NYC: South Asian Restaurants (Courtesy: New York City Department of Health and Mental Hygiene).

	BRONX	BROOKLYN	MANHATTAN	QUEENS	STATEN ISLAND	NYCTotal	% of total in NYC

**Indian**							
**GRADE A**	7	51	125	82	7	272	81%
**GRADE B**	1	8	5	12	1	27	8%
**GRADE C**	0	1	5	1	0	7	2%
**GRADE PENDING**	0	4	10	6	0	20	6%
**CLOSED**	0	0	3	0	0	3	1%
**TOTAL**	8	64	148	107	8	335	
**Bangladeshi**							
**GRADE A**	4	5	4	15	–	28	74%
**GRADE B**	0	0	2	3	–	5	13%
**GRADE C**	0	0	1	0	–	1	3%
**GRADE PENDING**	1	3	0	0	–	4	11%
**CLOSED**	0	0	0	0	–	0	0%
**TOTAL**	5	8	7	18	–	38	
**Pakistani**							
**GRADE A**	3	10	5	9	1	28	85%
**GRADE B**	0	1	0	2	0	3	9%
**GRADE C**	0	0	0	0	0	0	0%
**GRADE PENDING**	0	1	0	1	0	2	6%
**CLOSED**	0	0	0	0	0	0	0%
**TOTAL**	3	12	5	12	1	33	

**Table 2:** Critical violations in Restaurant Inspection and its possible implication for worker health and safety (Courtesy: New York City Department of Health and Mental Hygiene).

Violations	Implication on Worker Health and Safety

Live roaches present in facility’s food and/or non-food areas.	Cockroaches are sources of allergens in the workplace especially for asthmatics and people with conditions like Allergic Rhinitis or atopic allergic conditions; Potent allergens are released from the saliva secretions, cast skins, dead bodies of cockroaches. Cockroaches have been suspected to specific disease outbreaks; it can carry *Salmonella typhimurium, Entamoeba histolytica*, and the *poliomyelitis virus*
Filth flies or food/refuse/sewage-associated (FRSA) flies present in facility’s food and/or non-food areas. Filth flies include house flies, little house flies, blow flies, bottle flies and flesh flies. Food/refuse/sewage-associated flies include fruit flies, drain flies and Phorid flies.	Housefly (*Musca domestica*)’s close association with people, its abundance, and its ability to transmit disease make it a great threat to human. Each housefly can easily carry more than 1 million bacteria on its body. Some of the disease-causing agents transmitted by houseflies are *Shigella spp*. (dysentery and diarrhea = shigellosis), *Salmonella spp*. (typhoid fever), *Escherichia coli*, (traveler’s diarrhea), and *Vibrio cholerae* (cholera). These organisms can be carried on the fly’s tarsi or body hairs, and frequently these are regurgitated onto food when the fly attempts to liquefy it for ingestion. Acute gastrointestinal problems can be related to these conditions and employees can become reservoir for outbreaks of these deadly diseases.
Evidence of mice or live mice present in facility’s food and/or non-food areas.	Rodent-associated diseases affecting humans include *plague, murine typhus, leptospirosis, rickettsial pox, and rat-bite fever*. Animal bites can cause lacerated wounds with the potential for secondary bacterial infections. Mice infested workplaces are hazardous for employees. Allergies to vermins can also potentially cause or exacerbate one’s pulmonary, upper respiratory tract and skin conditions (allergic dermatitis)
Hot food item not held at or above 140°F. Cold food item held above 41°F (smoked fish and reduced oxygen packaged foods above 38°F) except during necessary preparation.	*These conditions can cause bacterial growth in the meat, poultry, eggs, seafood, dairy products. These* can potentially cause the outbreak of gastroenteritis or other acute GI related conditions for employees consuming this food. Sub-standard heating and cooling services for food storage/preparation point to a lack of other provisions and necessary equipment to ensure a safe workplace.
Sanitized equipment or utensil, including in-use food dispensing utensil, improperly used or stored No facilities available to wash, rinse and sanitize utensils and/or equipment. Wiping cloths soiled or not stored in sanitizing solution.	Musculoskeletal injuries and concussion related to utensils falling over. Sprain/strain and slip and fall injuries from cluttered tight spaces with limited maneuverability. Lack of space to store equipment points to confined spaces and possible fire hazards. Improper storage in the dishwashing area can lead to slips, trips and falls. Inadequate space for dishwashing may lead to repetitive strain traumatic injuries
Food not protected from potential source of contamination during storage, preparation, transportation, display or service. Raw, cooked or prepared food is adulterated, contaminated, cross-contaminated, or not discarded in accordance with HACCP plan.	These conditions can cause bacterial growth in the meat, poultry, eggs, seafood, dairy products. These can potentially cause the outbreak of gastroenteritis or other acute GI related conditions if food is consumed by the employees.
Tobacco use, eating, or drinking from open container in food preparation, food storage or dishwashing area observed.	Indoor smoking in the restaurant can lead to tobacco related exposures and second-hand smoking to the workers. Can be a fire hazard.
Food Protection Certificate not held by supervisor of food operations.	Not displaying this certificate may indicate a behavior of ignorance, unawareness or willful disregard. Ensuring healthy and safe workplace is highly unlikely in such a workplace and underreporting of work injuries will be common.
Personal cleanliness inadequate. Outer garment soiled with possible contaminant. Effective hair restraint not worn in an area where food is prepared.	Non-adherence to the hygienic practices indicates lack of proper training of the employees to adopt basic handling practices. Employees are unlikely to have any training on workplace safety. In some cases, employees’ exhibiting of these behaviors could be related to anxiety, depression, or stress resulting from the extreme economic insecurity or uncertainty.
Food contact surface not properly washed, rinsed and sanitized after each use and following any activity when contamination may have occurred. Food worker does not use proper utensil to eliminate bare hand contact with food that will not receive adequate additional heat treatment.	Lack of adequate washing of the utensils or cleanliness indicates non- adherence to basic hygienic practices and may lead to a host of illnesses among employees.
Hand washing facility not provided in or near food preparation area and toilet room. Hot and cold running water at adequate pressure to enable cleanliness of employees not provided at facility. Soap and an acceptable hand-drying device not provided.	Sink accessibility promotes handwashing. Similarly, glove accessibility is related to glove use. Some workers may not know that food needs to be protected from dirty hands. Glove use may need less handwashing. Workers wearing of gloves may not be a common practice in these ethnic restaurant establishments.

The NY Health Department inspects about 24,000 restaurants a year to monitor compliance with City and State food safety regulations [9]. The Health Department conducts unannounced inspections of restaurants at least once a year and since 2010, it has required restaurants to post letter grades showing sanitary inspection results where they can easily be seen by customers. Inspectors check for food handling, food temperature, personal hygiene, facility and equipment maintenance and vermin control among other safety protocols. Restaurants with a score between 0 and 13 points are ranked A, those with 14 to 27 points receive a B and those with 28 or more a C. These results are posted on the Health Department’s website as well and we have used this publicly and readily available data-source as a proxy or surrogate measure (Table 1).

We have compiled inspection data on Indian, Pakistani and Bangladeshi restaurants in NYC. We have used 1) overall restaurant grade and 2) specific violations as means of assessing workplace health and safety.

These overall grade findings suggest that 19% of the Indian, 26% of Bangladeshi, and 15% of Pakistani restaurants did not achieve grade A in these inspections. These suggest that workers in about 20% of these restaurants more than likely work in a relatively hazardous or unhygienic working conditions. If these restaurants are not attentive to quality or cleanliness of their core business – preparation and serving of food, it is quite likely that they are not paying adequate attention to protect or promote their workers’ health, safety and wellbeing.

In the second approach, we prepared an inventory of most common critical violations as reported in these inspection reports and examine here if these violations may have an implication for workers’ health and safety (Table 2).

In this report, we examine how regulatory or other business operations measures may be used for health surveillance. There is evidence correlating quality of customer service with workplace health and safety practice in some industries. In healthcare industry, there is evidence that organizations with stronger patient safety culture are also safer workplaces for its employees. In the restaurant business, we have explored using restaurant inspection grade as a proxy measure for employee safety and working conditions. Learning more about the restaurant inspection method, criteria set, and grading mechanism used may provide us with additional insight in understanding health and wellness of restaurant employees. We already know that migrant workers with lower socioeconomic conditions are less forthcoming and engaged in reporting their working conditions, hazards, injuries and illnesses and this kind of proxy measure as explored in this study may prove to be a useful and practical measure for such an industry.

We need to further validate this proposed alternative surveillance tool as this grading system has evolved over the last several years. There appears to be better understanding and appreciation of the grading system since its implementation in 2011, and there has been a continuous effort to improve the accuracy of this system. Incorporating a few worker health and safety measures in the current grading system can be a very useful next step.

There is a great need to develop workplace health and safety surveillance systems for small businesses to systematically understand the cause, nature and severity of injuries and illness. Surveillance data would identify high risk worker groups within these restaurants, as looking at time trends would help in evaluating interventions. We need a better understanding of work related injuries and illnesses in these small ethnic restaurants and establishments. We need to understand the utilization pattern of existing worker’s compensation system for the treatment and management of health conditions by the injured workers. As a next step, we may look forward to developing a customized program geared towards the prevention and remediation of work related injuries in these ethnic restaurant environments. Occupational healthcare providers need to become more culturally sensitive in their delivery of care. Topics on immigrant health should be included in formal training programs and curriculum. Occupational health professionals should be particularly aware of immigrant workers in the informal sector or small businesses who may be at a disadvantage due to their limited understanding and reporting of occupational hazards and disenfranchised from the formal legal, social safety and health care delivery system.
